# Differential dynamics of histone H3 methylation at positions K4 and K9 in the mouse zygote

**DOI:** 10.1186/1471-213X-4-12

**Published:** 2004-09-21

**Authors:** Konstantin Lepikhov, Jörn Walter

**Affiliations:** 1 Department of Natural Sciences – Technical Faculty III FR 8.3, Biological Sciences, Institute of Genetics/Epigenetics, University of Saarland, Saarbrücken, Germany

## Abstract

**Background:**

In the mouse zygote the paternal genome undergoes dramatic structural and epigenetic changes. Chromosomes are decondensed, protamines replaced by histones and DNA is rapidly and actively demethylated. The epigenetic asymmetry between parental genomes remains at least until the 2-cell stage suggesting functional differences between paternal and maternal genomes during early cleavage stages.

**Results:**

Here we analyzed the timing of histone deposition on the paternal pronucleus and the dynamics of histone H3 methylation (H3/K4mono-, H3/K4tri- and H3/K9di-methylation) immediately after fertilization. Whereas maternal chromatin maintains all types of histone H3 methylation throughout the zygotic development, paternal chromosomes acquire new and unmodified histones shortly after fertilization. In the following hours we observe a gradual increase in H3/K4mono-methylation whereas H3/K4tri-methylation is not present before latest pronuclear stages. Histone H3/K9di-methylation is completely absent from the paternal pronucleus, including metaphase chromosomes of the first mitotic stage.

**Conclusion:**

Parallel to the epigenetic asymmetry in DNA methylation, chromatin modifications are also different between both parental genomes in the very first hours post fertilization. Whereas methylation at H3/K4 gradually becomes similar between both genomes, H3/K9 methylation remains asymmetric.

## Background

It is now generally accepted that the properties of a particular DNA sequence in cells are not solely defined by the nucleotide sequence itself, but by "epigenetic" modifications as well. Epigenetic modifications imply the methylation of cytosine residues in CpG dinucleotides and covalent modifications of core histones. These modifications allow for flexible, but heritable at the same time, reprogramming of the genome.

In histone H3 five lysine residues can be methylated (K4, K9, K27, K36 and K79) [1]. Methylation at K4 and K9 play opposite roles in structuring repressive or accessible chromatin domains, with K4 methylation associated with transcriptionally active chromatin and K9 methylation with inactive chromatin in higher eukaryotes [2]. In addition, these lysine residues can be mono-, di- or tri-methylated, which contributes to the distinct qualities of H3/K4 and H3/K9 methylation. Similar to H3/K9 methylation, DNA methylation is associated with silenced chromatin and there appeared to be an interplay between the two epigenetic modifications. It is still an open question whether DNA methylation directs H3/K9 methylation or other way around, for both scenarios the experimental evidences do exist [3,4]. It recently has been shown that in mammalian cells the maintenance DNA methyltransferase DNMT1 is associated with proteins involved in chromatin reprogramming, including histones deacetylases, and is required for the establishment of H3/K9 methylation [5]. Various experimental data suggest that the DNA methylation causes multiple changes in local nucleosomes, such as deacetylation of histones H3 and H4, prevents H3/K4 methylation and induces H3/K9 methylation [6].

The fertilization of mouse egg causes dramatic changes in organization of both paternal and maternal genomes. Initially arrested in metaphase II oocyte completes the meiosis, forming haploid maternal pronucleus and extruding the second polar body. The densely packed with protamines sperm DNA decondences, protamines get exchanged by histones and DNA undergoes active demethylation. The demethylation in the early mouse zygote occurs asymmetrically on paternal DNA and affects different classes of repetitive and single copy sequences, but not the control regions of imprinted genes [7,8]. Previous studies have shown the exclusive localization of methylated H3/K9 in maternal pronucleus of the mouse zygote, which additionally marks the epigenetic asymmetry between maternal and paternal pronuclei [9-11].

Here we examine time dependent changes of chromatin structure in the mouse zygote, focusing on the dynamics of the acquisition of histones in the paternal pronucleus and methylation status of histone H3 at positions K4 and K9.

## Results and discussion

In order to obtain mouse zygotes at different stages of development and to provide the precise timing for fertilization we used *in vitro *fertilization of mature mouse oocytes. Histones and methylated H3/K4 and H3/K9 were detected by using indirect immunofluorescence. In our experiments we used antibodies, which specifically recognize mono- or tri-methylated forms of H3/K4, and di-methylated H3/K9. The zygotes were analyzed after 3, 5, 8, 10, 12 and 18 hours incubation of mature oocytes with capacitated sperm from donor males. After 18 hours most of embryos were found to be at metaphase and some already at telophase stage of the first mitotic division. Even using *in vitro *fertilization, the obtained zygotes are not completely synchronous in their development and it is more appropriate to use PN stages classification, which is based on the morphological changes of both pronuclei [8,12].

### Appearance of histones on paternal chromosomes

**Figure 1:**
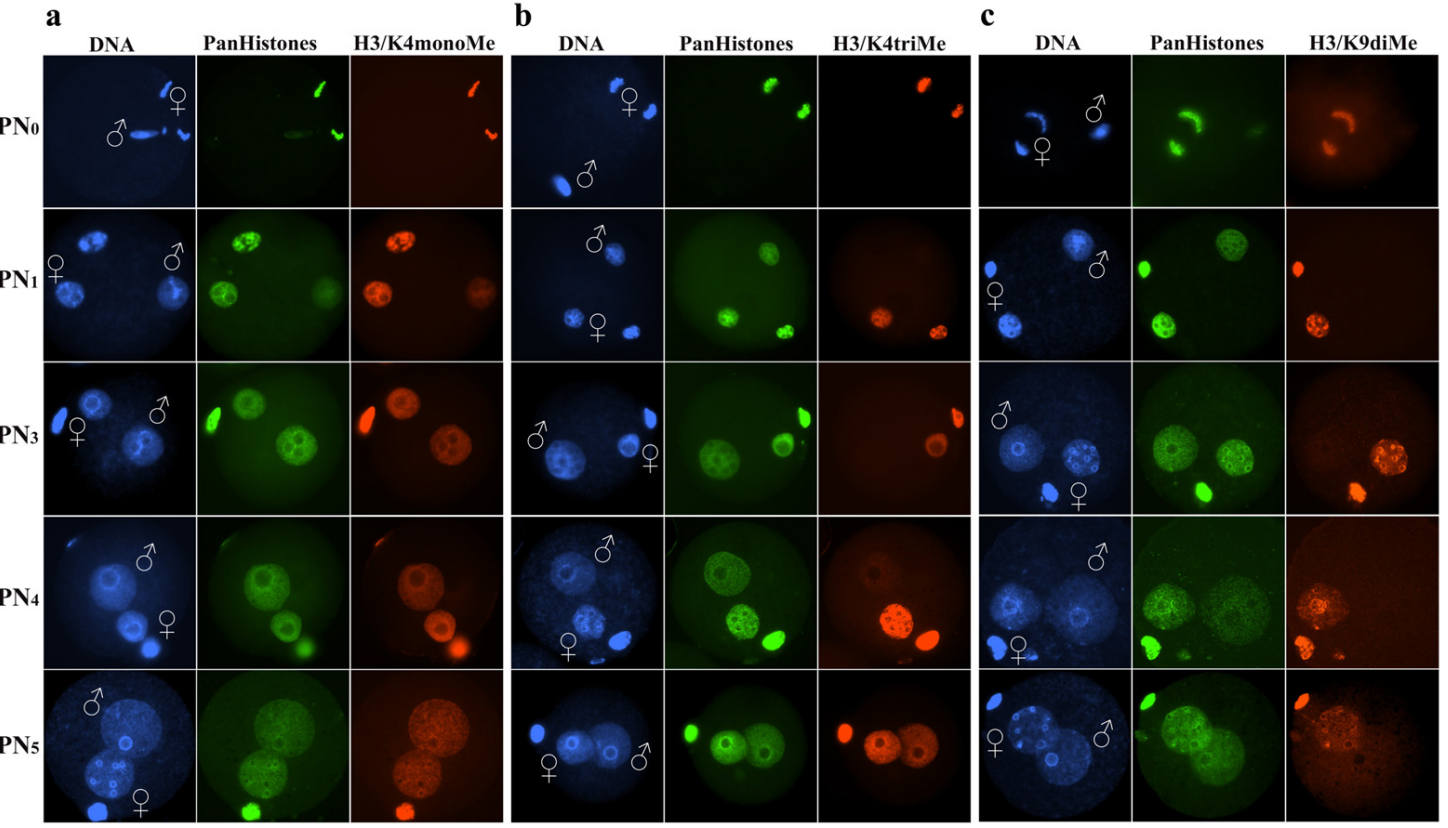
**Dynamic changes in chromatin of zygotes at different pronuclear stages. **DNA is visualized by DAPI (blue colour) staining. Mouse monoclonalanti PanHistones antibodies were detected by fluorescein conjugated anti-mouse secondary antibodies (green colour). Specific rabbit polyclonal antibodies, recognizing H3/K4monoMe (a), H3/K4triMe (b) or H3/K9diMe (c) were detected by Rhodamine Red-X conjugated anti-rabbit secondary antibodies (red colour).

We performed the immunostaining against core histones (anti-PanHistone antibodies) in all the stages tested in combination with antibodies, recognizing the specific methylated forms of histone H3. This served as a positive control for the immunostaining procedure and allowed us to follow the dynamics of histone acquisition in the paternal pronucleus. Histones were first detected shortly after the penetration of sperm into the oocyte and the beginning of the decondensation of sperm chromatin. According to PN stages classification we could clearly detect histones on paternal pronucleus at late PN_0_/early PN_1 _stages (approx. 3–5 hours p.f.), exactly when the global demethylation starts [8] (Fig. 1).

### Dynamic changes in H3/K4 methylation in paternal genome

**Figure 2:**
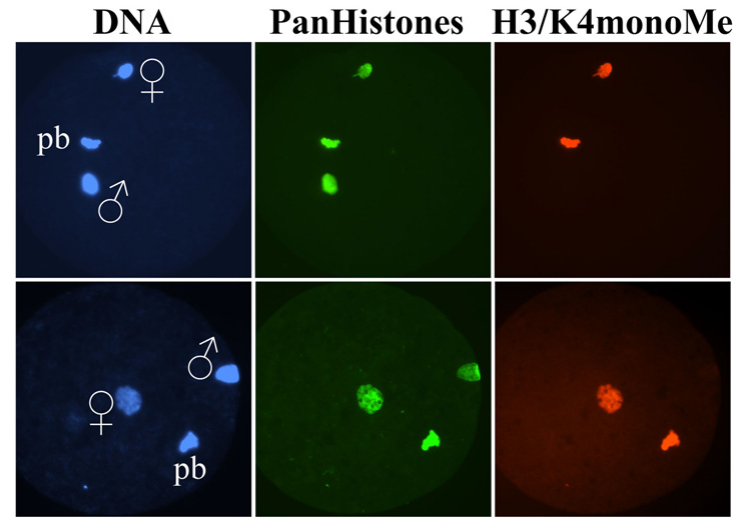
**Distribution of histones and H3/K4monoMe in the zygotes at late PN_0 _stage. **At this stage histones (green signal) are detectable in both male (♂) and female (♀) pronuclei, whereas H3/K4monoMe (red signal) is only detectable in female pronucleus and polar body (pb).

**Figure 3:**
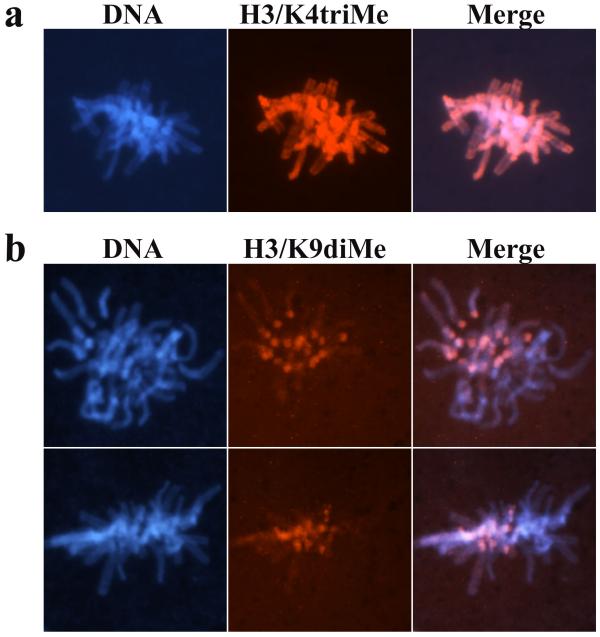
**Distribution of H3/K4triMe and H3/K9diMe in metaphase chromosomes during the latter portion of the first cell cycle. **(a) Distribution of H3/K4triMe. Paternally and maternally derived chromosomes show equal staining pattern along the whole length of chromosomes. (b) Distribution of H3/K9diMe. This type of modification is not detectable on paternal chromosomes and in maternal chromosomes is mostly associated with centromeres.

Probing the mouse zygotes at different stages with antibodies specifically recognizing either mono- or tri-methylated H3/K4 revealed that these types of modifications are associated with maternal genome through all zygotic stages, including mature oocyte and seem to be rather ubiquitous (Fig. 1a,1b). As for the paternal pronucleus – we detect the appearance of H3/K4mono-methylation in the beginning of PN_1 _(approx. 5 hours p.f.) stage (Fig. 1a), only slightly delayed compared to the appearance of core histones (Fig. 2). By PN_3 _– PN_4 _stages both paternal and maternal pronuclei show equal staining intensity. This indicates that H3/K4 specific histone methyltransferase, possibly Set9 [13], is quite active in the early zygote and methylates histone H3 after it is incorporated into the nucleosomes. In contrast to that, it has been shown recently that H3/K9 specific histone methyltransferase is inactivated immediately after the fertilization by yet unknown active mechanism, which involves *de novo *synthesis of some specific factors [11]. H3/K4tri-methylation becomes detectable later, starting from PN_4 _stage (approx. 8–10 hours p.f.) and the difference in antibodies staining intensity between paternal and maternal pronuclei becomes indistinguishable in the last pronuclear stage PN_5 _(approx 12 hours p.f.) (Fig. 1b) and in metaphase stage of first mitosis approximately 16 hours p.f. (Fig. 3a). The fact that H3/K4 first becomes mono-methylated and several hours later tri-methylated suggests progressive methylation of histone H3 at lysine 4. We also suggest that histone H3 gets incorporated into the nucleosomes being unmethylated and then undergoes methylation because we observe first the appearance of histones and then H3/K4mono-methylation. In contrast to that – acetylation of histones H3 and H4 happens before they are incorporated into the nucleosomes, and after the nucleosome assembly they can get deacetylated by histone deacetylases (HDACs) whenever required [14]. But no histone demethylase has been found so far.

### H3/K9 methylation but not H3/K4 defines the genomes asymmetry in the mouse zygote

In order to compare the patterns of H3/K4 and H3/K9 methylation we performed the immunostaining of mouse zygotes using antibodies, which recognize di-methylated H3/K9. Our results are in the agreement with earlier observations that H3/K9 methylation is only attributed to the maternal genome and is completely absent from the paternal [9-11] (Fig. 1c, Fig. 3b). In normal somatic cells the absence or disruption of H3/K9 methylation leads to the chromosome instability and affects chromosomes segregation during mitosis [15]. Therefore the absence of H3/K9 methylation on paternal chromosomes is rather surprising and compromises its role in chromosomes segregation. The epigenetic asymmetry between paternal and maternal genomes is observed till 2-cell stage and is characterized by low levels of DNA methylation and H3/K9 methylation in paternal genome [8,10,11,16]. In case with H3/K4 methylation – the asymmetry is observed only in the beginning of the zygotic development and is indistinguishable in the metaphase stage of the first mitotic division (Fig. 3a). Recent data from Liu *et al*. suggest that H3/K9 methylation does not depend on DNA methylation [11], but it is only paternal DNA which gets demethylated in the mouse zygote and at the same time it does not have detectable H3/K9 methylation. According to data published by Santos *et al*. [17,18] DNA demethylation starts at PN_1 _stage, *i.e. *at a time when we first observe the appearance of H3/K4mono-methylation (PN_1 _stage, Fig. 1a), and is completed at PN_3 _stage when H3/K4mono-methylation in paternal pronucleus reaches approximately the same level as in the maternal (Fig. 1a). This fact is raising the question if such a coincidence might indicate that DNA demethylation and the establishment of H3/K4 methylation are interdependent. Demethylation of paternal DNA upon the fertilization is not a universal phenomenon for mammalian species. In bovine zygote paternal DNA becomes only partially demethylated, while in sheep and rabbit zygotes the demethylation is hardly detectable [17,18]. The analysis of chromatin modification in early zygotes of these species might help to get an answer if DNA demethylation depends on, or is directed by the specific chromatin modifications.

## Conclusions

Unlike H3/K9 methylation, methylation of H3/K4 is not attributed only to the maternal genome but appears shortly after the acquisition of histones by paternal pronucleus. The methylation of H3/K4 is progressive and by first mitotic division reaches approximately same level as in maternal genome.

## Methods

### *In vitro *fertilization of mouse oocytes

As sperm and oocytes donors we used (C57BL/6 X CBA)F_1 _mice. Mature oocytes were collected 14 hours post human chorionic gonadotropin injection according to standard procedures [19]. Sperm isolation and *in vitro *fertilization (IVF) procedures were performed as described in [20]. Briefly: the sperm was isolated from cauda epididimus of donor males and capacitated in pre-gassed HTF medium for 1,5 hours. Isolated oocytes in cumulus cell mass were placed into 100 μl drop of HTF medium with capacitated sperm and incubated in CO_2 _incubator for 3, 5, or 8 hours. For longer incubation time the oocytes were incubated with sperm in HTF medium for 8 hours and then transferred into the drop of pre-gasses and pre-warmed M16 medium and incubated further for 2, 4 or 10 hours.

### Immunofluorescence staining

After the removal of zona pellucida by treatment with Acidic Tyrode's solution fertilized oocytes were fixed for 20 min in 3.7% paraformaldehyde in PBS, and permeabilized with 0.2% Triton X-100 in PBS for 10 min at room temperature. The fixed zygotes were blocked overnight at 4°C in 1% BSA, 0.1% Triton X-100 in PBS. After blocking the embryos were incubated in the same solution with either anti PanHistones (mouse polyclonal, Roche), anti mono-methyl H3/K4 (rabbit polyclonal, Abcam), anti tri-methyl H3/K4 (rabbit polyclonal, Abcam) or anti di-methyl H3/K9 (rabbit polyclonal, a gift from T. Jenuwein [21] antibodies at room temperature for 1 hour, followed by several washes and incubation for 1 hour with anti-mouse secondary antibodies coupled with fluorescein (Sigma-Aldrich), and anti-rabbit secondary antibodies coupled with Rhodamine Red-X (Jackson ImmunoResearch Laboratories Inc.). After final washes the zygotes were placed on slides and mounted with a small drop of Vectashield (VectorLab) mounting medium containing 0.5 μg 4,6-diamino-2-phenylindole (DAPI). At least 20 zygotes have been analyzed for each stage of zygotic development.

### Immunofluorescence microscopy

The slides were analyzed on Zeiss Axiovert 200 M inverted microscope equipped with the fluorescence module and B/W digital camera for imaging. The images were captured, pseudocoloured and merged using AxioVision software (Zeiss).

## Authors' contributions

KL conducted the experimental part of the work and co-wrote the manuscript. JW coordinated the study and co-wrote the manuscript. All authors read and approved the final manuscript.
